# Pendulum swings from hypo- to hyperthyroidism: thyrotoxicosis after severe hypothyroidism after neck irradiation in a patient with a history of Hodgkin's lymphoma

**DOI:** 10.1186/1756-6614-8-S1-A18

**Published:** 2015-06-22

**Authors:** Krzysztof Lewandowski, Katarzyna Dabrowska, Jacek Makarewicz, Andrzej Lewinski

**Affiliations:** 1 Department of Endocrinology and Metabolic Diseases, Polish Mother’s Memorial Hospital Research Institute, The Medical University of Lodz, Lodz, Poland; 2 Department of Nuclear Medicine and Oncological Endocrinology, Zgierz District Hospital, Zgierz, Poland

## Case presentation

A 27-year old female presented with clinical and biochemical thyrotoxicosis (TSH 0.01 µIU/mL (ref. range: 0.27-4.2 µIU/mL); FreeT4 1.58 ng/dL (ref. range 0.98-1.63 ng/dL); FreeT3 4.56 pg/mL (ref. range 2.6-4.4 pg/mL). Clinical examination revealed tachycardia at about 100 beats/minute and no obvious goitre. Autoimmune profile was suggestive of Graves’ disease (anti-TSH-receptor antibodies (aTSHR) 16.69 IU/L (ref. 0-1.75), anti-thyroid peroxidase antibodies (aTPO) 1780 IU/mL (ref.: 0-34 IU/mL). The patient had a history of Hodgkin’s lymphoma, diagnosed and treated with chemo- and radiotherapy (including the neck) at the age of 18. At the age of 20 she developed severe hypothyroidism (TSH>100 µIU/ml), though with high titres of both aTPO (150 IU/mL) and aTSHR (37.56 IU/mL) antibodies. Thyroid function tests normalised after treatment with L-thyroxine (100 µg od). At the age of 26 she became “anxious” and experienced “heart palpitations”. She was found to have suppressed TSH, that remained suppressed even when the dose of L-thyroxine was reduced and then discontinued. After further four months she was found to have raised free T3 (see above). Thyroid scintigraphy revealed a normal and homogenous iodine uptake (41%). The patient responded very well to treatment with low dose thiamazole (10 mg od). As subclinical thyrotoxicosis persisted after discontinuation of thiamazole, she was eventually referred for treatment with radioiodine.

## Discussion

Thyroid dysfunction is one of the most common abnormalities seen after radiotherapy for Hodgkin's disease that includes the neck [1]. Primary hypothyroidism, the most common radiation-induced thyroid dysfunction, appears in 20–30% of patients who had therapeutic radiotherapy administered to the neck region, and usually occurs within the first 5 years after therapy (peak 2-3 years after treatment) [1]. Irradiation of the thyroid may also increase the risk of Graves' disease (relative risk 7.2–20.4%), or Graves' ophthalmopathy, thyroiditis, benign adenomas and thyroid cancer. The aetiology of radiation-induced thyroid dysfunction includes vascular damage, parenchymal cell damage and auto-immune reactions [1]. According to some authors, thyroiditis observed in Hodgkin’s disease may be the result of immune regulation disorders in Hodgkin’s disease [2]. Our case illustrates that after neck irradiation, severe hypothyroidism can be followed by thyrotoxicosis. In both situations, aTSHR were elevated. There are two types of aTSHR: thyroid stimulating antibody (TSAb) and TSH-stimulation blocking antibody (TSBAb). TSBAb blocks TSH-stimulation of the thyroid and causes hypothyroidism. TSAb stimulates the thyroid and causes Graves’ hyperthyroidism. In our opinion, change of thyreometabolic state is possible, because in this case there was a gradual switch from a TSH receptor blocking antibodies (TSBAb) into TSH receptor stimulating antibodies(TSAb). In some patients, TSAb and hyperthyroidism develop unexpectedly after hypothyroidism that is caused by TBAb [3]. Also, in some hypothyroid patients after irradiation of the neck with Hodgkin's disease developed hyperthyroiditism [4]. This shift in thyroid function occurs rarely [3,4]. A number of mechanisms may be involved in switching from TBAb to TSAb. Significantly, thyroxine treatment in some patients is associated with increased TSAb that in extreme cases might lead to development of hyperthyroidism in hypothyroid patients [3]. There are reports that after neck irradiation Graves’ disease may develop in patients receiving thyroxine, and 33% of the patients with Graves’ hyperthyroidism had received thyroxine before its onset [4]. Therefore thyroid hormone-replacement therapy in patients with hypothyroidism after irradiation of the neck does not eliminate risk of later thyroid abnormalities.
